# P033 Giant cell arteritis identified on biopsy from a neck lymph node dissection: an incidental finding

**DOI:** 10.1093/rap/rkag113.063

**Published:** 2026-11-03

**Authors:** Daniel Coulter, Claire Masih, Simon Rajendran, Suleman Khan

**Affiliations:** Ulster Hospital Dundonald, Belfast, United Kingdom; Ulster Hospital Dundonal, Belfast, United Kingdom; Ulster Hospital Dundonald, Belfast, United Kingdom; Ulster Hospital Dundonald, Belfast, United Kingdom

## Abstract

**Introduction:**

Large vessel giant cell arteritis (LV-GCA) has become a well-established condition due to advancements in vascular imaging in recent decades. It is commonly diagnosed by PET-CT, demonstrating increased glucose re-uptake in the walls of inflamed arteries, performed to investigate unexplained constitutional symptoms with raised inflammatory markers. Histology is most commonly utilised for diagnostic purposes to investigate cranial GCA via a temporal artery biopsy (TAB). We present an unusual case of LV-GCA identified incidentally in the soft tissue from a neck dissection performed as part of surgical removal of an oral squamous cell carcinoma.

**Case description:**

We describe a case of a 70-year-old female with a three-year history of polymyalgia rheumatica (PMR) diagnosed in primary care. She was receiving 5mg of prednisolone daily since October 2025, having previously been unable to wean off steroids completely due to the return of proximal pains on dose reductions without a corresponding rise in inflammatory markers, with the lowest dose achieved being 2mg daily. In December 2025, she noticed a swelling in her gum which was confirmed on biopsy as a squamous cell carcinoma of her posterior alveolus and cheek. Subsequent CT neck and chest and MRI neck with contrast showed no bony or lymphatic invasion and the cancer was staged as T2N0M0. She underwent a curative left partial maxillectomy and neck dissection in March 2026, with the soft tissue of the neck demonstrating thick-walled vessels with luminal narrowing and lymphohistiocytic inflammatory cell infiltrate extending transmurally with giant cell formation.

When reviewed by the rheumatology team in April, she reported intermittent headaches since she was a teenager, which were unchanged of late and not felt to be clinically relevant. She denied any systemic upset or symptoms suggestive of cranial-GCA or active PMR. Clinical examination was unremarkable, inflammatory markers normal (ESR 15mm/h, CRP 5mg/L) and ANCA antibodies negative. Her steroid dose remained at 5mg and a PET-CT was arranged, which showed circumferential activity at the thoracic aorta with prominent activity extending into both subclavian arteries, with no abnormalities in the carotid arteries or periarticularly at the hips or shoulders. Prednisolone was subsequently increased to 40mg and ultrasound of her temporal arteries performed three weeks after this increase in steroid therapy demonstrated normal temporal arteries with increased thickness of the axillary artery bilaterally. She remains clinically well with no clinical or biochemical features of vasculitis.

**Discussion:**

This patient’s presentation was unusual both due to the lack of any clinical or biochemical evidence supporting a diagnosis of large vessel vasculitis (LVV) and GCA being demonstrated incidentally in neck vessels biopsied for another reason. When the only supportive feature of a diagnosis of LV-GCA was histology, the case was discussed at the local rheumatology multidisciplinary team meeting and the decision was made not to commence high dose steroids due to the possibility this was a vasculitic mimic such as malignancy. The role of histology in the diagnosis of LVV is commonly restricted to a TAB. The 2018 European League Against Rheumatism (EULAR) guidelines recommend suspected cases of LVV should be confirmed by imaging or histology from a TAB, with the 2023 guidelines shifting the focus of diagnosis more towards imaging by suggesting TAB is only required in cases of inconclusive or negative imaging if pre-imaging clinical suspicion was high. A literature search has not identified any other cases of LV-GCA presenting as an incidental finding in the vessels present in the soft tissue of the neck on biopsy.

At the time of the initial PMR diagnosis the patient’s inflammatory markers were modestly elevated with a CRP of 25mg/L, however the lack of symptoms or raised inflammatory markers at initiation of high dose steroids make decisions regarding disease monitoring complex. The 2023 EULAR guidelines state imaging is not routinely recommended for patients in clinical and biochemical remission but it is not clear whether this should apply to patients who presented in this state. Monitoring plans for the next year are to be based on clinical assessment alongside checking inflammatory markers and consideration is being given to introducing tocilizumab alongside glucocorticoids, provided the maxillofacial team have no objections from a wound healing perspective.

**Key learning points:**

• This case raises a number of learning points relevant to the diagnosis of LVV and illustrates how close contact is often required between specialties, including primary care, to facilitate a diagnosis. Firstly, a biopsy result suggestive of giant cell arteritis even without supporting clinical features and from an atypical site should always be regarded as significant and warrants further investigation. The presence of both multinucleated giant cells and transmural inflammation on a TAB, as was seen in the 4mm neck vessel in this case, has 100% specificity for GCA. The decision whether to commence treatment whilst awaiting further investigations is likely to be influenced by the speed of availability of specialist imaging locally - in this case, there were three weeks between the rheumatology assessment and PET-CT being performed.

• Secondly, this case reinforces the role of PET-CT in cases of ‘atypical PMR’ where the patient has been unable to wean off steroids. Whilst it is well established to utilise PET-CT in PMR cases with ongoing symptoms and elevated inflammatory markers on reduction of steroids, this case demonstrates that PET-CT can be useful even when inflammatory markers are normal. The case also highlights the benefits of close contact between primary and secondary care when managing PMR, especially when treatment difficulties arise, which is advocated in the 2024 treat-to-target recommendations in giant cell arteritis and polymyalgia rheumatica.

• We hope that discussing this case with a range of colleagues at the conference will be a useful experience for all involved, giving us insight into whether others have also encountered the initial diagnostic conundrum we faced: histological GCA from an atypical site without supporting clinical evidence.

